# Comparative Analysis of Free Amino Acids and Nucleosides in Different Varieties of Mume Fructus Based on Simultaneous Determination and Multivariate Statistical Analyses

**DOI:** 10.1155/2020/4767605

**Published:** 2020-08-01

**Authors:** Jinmei Ou, Rui Wang, Xiaoli Li, Luqi Huang, Qingjun Yuan, Chengwu Fang, Deling Wu

**Affiliations:** ^1^Anhui University of Chinese Medicine, Heifei 230038, China; ^2^China Academy of Chinese Medical Sciences, Beijing 100700, China

## Abstract

Mume Fructus (MF) contains a variety of organic acids, free amino acids, and nucleoside components, and studies have not yet analyzed the relationship between the components of free amino acids and nucleosides with the varieties of MF. A rapid and sensitive method was established for simultaneous determination of 21 free amino acids and 9 nucleosides in MF by ultrafast liquid chromatography-mass spectrometry. The analysis was carried out on a Waters XBridge Amide column (100 mm × 2.1 mm, 3.5 *μ*m) with elution by the mobile phase of 0.2% aqueous formic acid (A) and 0.2% formic acid acetonitrile (B) at a flow rate of 0.2 mL/min with 1 *μ*L per injection. The column temperature was maintained at 30°C. The target compounds were analyzed by the positive ion multiple reaction monitoring (MRM) mode. The comprehensive evaluation of the samples was carried out by principal component analysis (PCA) and technique for order preference by similarity to an ideal solution (TOPSIS) analysis. Results showed the method could simultaneously determine 30 components in MF. The content of total analytes in six mainstream varieties was different, exhibited the order Nangao > Daqingmei > Zhaoshuimei > Yanmei > Shishengme > Baimei, and aspartic acid and adenosine were the most abundant amino acid and nucleoside. PCA and OPLS-DA could easily distinguish the samples, and 11 components could be chemical markers of sample classification. TOPSIS implied that the quality of Nangao and Daqingmei was superior to the other varieties. The results could provide a reliable basis for quality evaluation and utilisation of medicinal and edible MF.

## 1. Introduction

Mume Fructus (MF) is derived from the immature fruit of *Prunus mume* Sieb. et Zucc., named Wumei (in China), and is commonly used food and Chinese medicines, with astringing the lung to stop cough, relieving diarrhea with astringents, removing toxicity for detumescence, promoting fluid relieving thirst, and calming *Ascaris* [1, 2]. Modern pharmacological studies have shown that it has antibacterial, anti-Alzheimer, antiulcer, antitumor, antivirus, and other biological activities [3–6]. For its important medicinal and edible processing value, MF is widely cultivated in China [7, 8], and the medicinal varieties are mostly distributed in Southwest areas of Sichuan and Yunnan provinces. In the preliminary investigation, some edible varieties were mixed as medicinal varieties, and the differences in appearance and internal components of varieties brought many problems to the identification and quality evaluation of MF [9].

Phytochemical studies have revealed that MF contains various components, including organic acids, amino acids, nucleotides, terpenoids, inorganic elements, volatile components, and polysaccharides [2, 10]. Organic acids are known as bioactive constituents, and amino acids and nucleotides also play synergistic roles with organic acids in the clinical effects of MF [11, 12]. Amino acids are the basic building blocks of proteins which are required for human nutrition [13, 14]; *L*-methionine, *L-*threonine, *L*-arginine, and glycine have beneficial effects on intestinal flora; the conclusion may explain the obvious antidiarrhea effect of MF [15]. Nucleosides have important physiological functions, such as adenosine can treat angina pectoris, myocardial infarction, cerebrovascular disorders, and other diseases [16]. Scholars have used the components of amino acids and nucleosides to evaluate the quality of medicines and edible medicinal plants [17–19], and the results showed that differences in the composition and contents of amino acids and nucleosides could be used as biomarkers to characterize the quality of medicines and food. Until now, few studies have focused on the components of amino acids and nucleosides in MF; the methodologies of the amino acid analyzer and PITC-HPLC methods mainly focus on quantification [20, 21] and have some deficiencies in resolution and separation time.

In the current study, an ultrafast liquid chromatography-mass spectrometry (UFLC-MS/MS) method was developed for the determination of 21 amino acids and 9 nucleosides in MF, and then the contents in six mainstream varieties were compared and analyzed by the multivariate statistical analysis method (including principal component analysis (PCA) [22, 23] and technique for order preference by similarity to an ideal solution (TOPSIS) method [24, 25]). The proposed method can provide theoretical basis for the quality evaluation and comprehensive utilisation of MF and other edible medicines.

## 2. Materials and Methods

### 2.1. Chemicals, Reagents, and Materials

Thirty chemical standards were used: *L*-isoleucine (1), *L*-histidine (2), *L*-phenylalanine (3), *L*-leucine (4), *L*-glutamic acid (5), *L*-tyrosine (6), *L*-alanine (7), *L*-cystine (8), *L*-threonine (9), *L*-methionine (10), *L*-proline (11), *L*-aspartic acid (12), *L*-asparagine (13), *L*-serine (14), glycine (15), *L*-valine (16), *L*-arginine (17), *L*-hydroxyproline (18), *L*-tryptophan (19), *L*-lysine hydrochloride (20), *γ*-aminobutyric acid (21), uracil (22), adenine (23), cytidine (24), inosine (25), thymidine (26), guanosine (27), adenosine (28), uridine (29), and hypoxanthine (30). The purity of all standard components was ≥98%. The structures of these standard substances are shown in Figure S1. Among them, *L*-isoleucine, *L*-histidine, *L*-phenylalanine, *L*-leucine, *L*-glutamic acid, *L*-tyrosine, *L*-alanine, *L*-cystine, *L*-threonine, *L*-methionine, and *L*-proline were purchased from the National Institutes for Food and Drug Control (Beijing China). The remainder were obtained from Shanghai Yuanye Biotechnology (Shanghai, China). Chromatography-grade methanol and acetonitrile were purchased from Merck (Darmstadt, Germany). Ultrapure water was produced by the Milli-Q purification system (Millipore, MA, USA).

These samples were collected from May to July 2018 in various production areas. Then, they were dried at low temperature, nucleated, and crushed through a sieve (60-mesh), dried, and stored under constant weight for testing. The botanical origins of the samples were identified by Chengwu Fang. Voucher specimens have been deposited in the Chinese Medicine Resource Centre, Anhui University of Chinese Medicine (Anhui, China). Information on the samples collected is listed in Table 1.

### 2.2. Preparation of Standard Solutions

**Figure 1:**
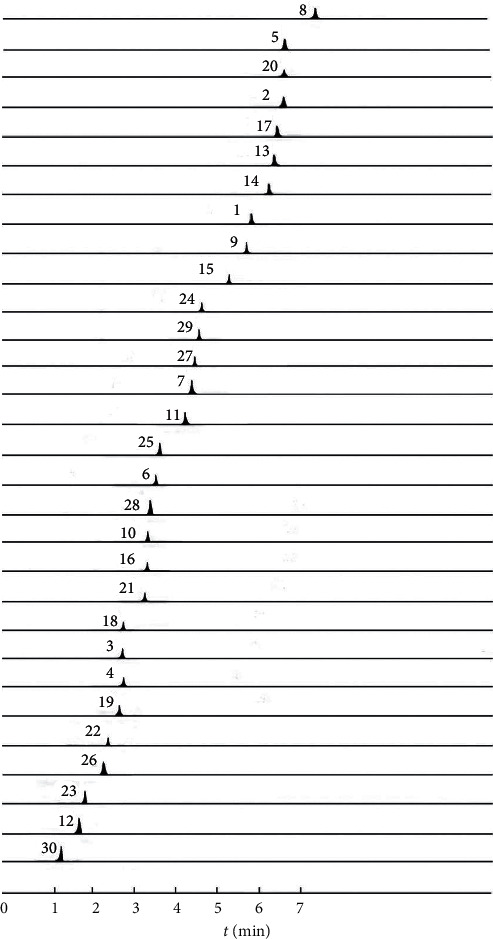
Multiple reaction monitoring (MRM) chromatograms of 30 compounds.

Thirty standard substances were prepared by dissolving in ultrapure water, and their concentrations (in mg/mL) were as follows: (1) 0.17, (2) 0.62, (3) 0.15, (4) 0.22, (5) 0.12, (6) 0.10, (7) 0.25, (8) 0.15, (9) 0.40, (10) 0.36, (11) 0.33, (12) 4.2, (13) 1.84, (14) 0.36, (15) 0.14, (16) 0.40, (17) 0.45, (18) 0.16, (19) 0.10, (20) 0.15, (21) 1.5, (22) 0.50, (23) 0.11, (24) 0.21, (25) 0.10, (26) 0.20, (27) 0.12, (28) 0.20, (29) 0.50, and (30) 0.13. Mixed standard stock solution containing 30 standard substances was serially diluted with ultrapure water to the required concentration for establishment of calibration curves. All solutions were stored at 4°C and then passed through a 0.22 *μ*m membrane. The typical chromatograms of analytes and the sample are presented in Figure 1.

### 2.3. Preparation of Sample Solutions

Accurately weighed powder (1.0 g) was extracted by ultrasonication with 20 mL of ultrapure water for 30 min and cooled at normal temperature. The same solution was used to replenish the extraction system upon solvent loss due to volatilisation. The mixture was centrifuged at 10,000 rpm for 5 min and passed through the 0.22 *μ*m membrane before analyses.

### 2.4. UFLC-MS/MS Instrumentation and Conditions

Samples were analyzed using an UFLC system (Shimadzu, Kyoto, Japan) with a triple quadrupole-linear ion trap mass spectrometer (QUAD-4500; AB Sciex, Framingham, MA, USA). An XBridge Amide column (100 mm × 2.1 mm, 3.5 *μ*m; Waters, Milford, MA, USA) was used for chromatographic isolation. The mobile phase consisted of 0.2% aqueous formic acid (A) and 0.2% formic acid acetonitrile (B) at a velocity of 0.2 mL/min. Gradient elution was 0–2.5 min: 15% A; 2.5–5 min: 15–50% A; 5–7 min: 50% A; 7-8 min: 50–15% A; and 8–11 min: 15% A. The column temperature was maintained at 30°C, and the injection volume was 1 *μ*L.

The ESI-MS spectra were acquired in the positive ion multiple reaction monitoring (MRM) mode with the nebuliser pressure 5.5 kV; gas temperature 550°C; curtain-gas pressure 241.3 kPa (35 psi); gas-1 pressure 379.2 kPa (55 psi); and gas-2 pressure 379.2 kPa (55 psi). Simultaneously, detection of ion pairs, cluster voltage (DP), and collision voltage (CE) was optimized. All MS data were analyzed by Analyst 1.6.2 (AB SCIEX). The optimized parameters for MS for the 30 target components are shown in Table 2.

### 2.5. Method Validation

The proposed method was validated, including linearity, range, the limit of detection (LOD), the limit of quantitation (LOQ), precision, repeatability, stability, and recovery. Under the present chromatographic conditions, the LODs and LOQs were obtained on the response of each regression equation at signal-to-noise ratios (S/N) of 3 and 10, respectively. The intra- and interday precision were investigated by analysing the 30 analytes in six replicates within one and consecutive three days. The repeatability was ensured by preparing and analysing six independent sample solutions from sample S10. The stability of the samples was verified by analysing solution of sample S10 at 0, 2, 4, 8, 12, and 24 h. All variations were expressed by the relative standard deviation (RSD). The recovery test was performed to evaluate the accuracy of the above methods. The amount of 30 standards was added to six accurately weighed samples of S10 (1.0 g) and then extracted, processed, and quantified orderly, and the average recovery rate and RSD were calculated.

### 2.6. Multivariate Statistical Analyses

Principal component analysis (PCA) is used to visualize the similarity or difference in multivariate data. It is a method of transferring multiple variables through linear transformation to select fewer important variables. SPSS23.0 was used to evaluate the variation of 30 components in MF samples. OPLS-DA supervised by SIMCA-p 14.1 was conducted with data of 30 analytes to discover different chemical compositions of each sample.

TOPSIS is used commonly in analyses of multiple-objective decisions. It ranks a limited number of evaluation objects according to their proximity to the ideal target. By calculating the best and worst indices of samples, we introduced a method to evaluate the quality of samples based on the content of 30 analytes.

The data matrices of 21 amino acids and 9 nucleoside components in samples were normalized, and the corresponding matrices were established to calculate the *Z*_*ij*_ value:

(1) $$\begin{array}{c} Z_{ij}=\frac{X_{ij}}{\sqrt{\sum_{i=1}^{n}X_{ij^{2}}}}, \end{array}$$

where *X*_*ij*_ represents the value of the *i* evaluation object on the *j* index and *Z*_*ij*_ represents the value of the *i* evaluation object after normalization on the *j* index.

The optimal vector (*Z*^+^) and the worst vector (*Z*^−^) consist of the maximum value and the minimum value of each column element. The distance from each evaluation object to *Z*^+^ and *Z*^−^ is calculated. *D*_*i*_^+^ and *D*_*i*_^−^ represent the distances of each evaluation object from *Z*^+^ and *Z*^−^, respectively:

(2) $$\begin{array}{c} D_{i}^{+}=\sqrt{\sum_{j=1}^{n}{\left(z_{ij}-z_{j}^{+}\right)}^{2}},\\ D_{i}^{-}=\sqrt{\sum_{j=1}^{n}{\left(z_{ij}-z_{j}^{-}\right)}^{2}}. \end{array}$$

The relative closeness to the ideal object (*C*_*i*_) is calculated, and *C*_*i*_ values are compared to rank alternatives. When *C*_*i*_ is between 0 and 1, closer to 1, the evaluation object is close to the optimal level; when it is closer to 0, the evaluation object is close to the worst level:

(3) $$\begin{array}{c} C_{i}=\frac{D_{i}^{-}}{D_{i}^{-}+D_{i}^{+}}. \end{array}$$

## 3. Results and Discussion

### 3.1. Sample Preparation Optimization

In order to obtain quantitative results, the extraction method was to be optimized. The extraction method adopted ultrasound extraction with water according to [26]. And the solvent volume (10, 20, 30, and 40 mL) and extraction time (10, 20, 30, and 40 min) were examined. The results showed that the optimal extraction method was ultrasonic treatment at room temperature for 30 min with 20 mL water as the solvent according to characteristic peak intensity.

### 3.2. Optimization of UFLC-MS/MS Conditions

Previous studies [16, 17] reported that the positive ion modes presented higher sensitivity and intensity in examining components of amino acids and nucleosides than negative ion modes, so the ESI+ mode was adopted in the study. Most components obtained intensive product ions by optimization of the MS parameters; just adenine and hypoxanthine were too low to be detected.

Chromatographic conditions were optimized. Different mobile phases (water/methanol; 0.1% aqueous formic acid/methanol; 0.1% aqueous formic acid/acetonitrile; and 0.2% aqueous formic acid/0.2% formic acid acetonitrile) were investigated. We found that acetonitrile had better separation efficiency than that of methanol. Upon addition of 0.2% aqueous formic acid into acetonitrile and water, the shape of the chromatographic peak and degree of separation were better, and the tail of the chromatographic peak was reduced. Furthermore, a flow rate of 0.1–0.5 mL/min, injection volume of 1–5 *μ*L, and column temperature of 30–40°C were studied to obtain rapid and reliable separation. As a result, the analysis was carried out on a Waters XBridge Amide column (100 mm × 2.1 mm, 3.5 *μ*m) with elution by mobile phase of 0.2% aqueous formic acid (A) and 0.2% formic acid acetonitrile (B) at a flow rate of 0.2 mL/min with 1 *μ*L per injection. The column temperature was maintained at 30°C, respectively.

### 3.3. Method Validation

Validations of the method are shown in Table 3. All calibration curves showed good linearity regressions (*R*^2^ > 0.9990) within the determination range. The LODs and LOQs of the 30 compounds were estimated to be in the range of 0.14–5.00 ng/mL and 0.18–16.67 ng/mL, respectively. They should provide satisfactory sensitivity for all analytes. The RSD values of intraday, interday, repeatability, and stability tests of the 30 compounds were 1.28%–3.29%, 1.78–3.23%, 1.74%–2.95%, and 1.44%–2.92%, respectively. The average recovery rates were 95.02–104.94%, with the RSD values of 2.01–3.89%, and the slope ratio of the matrix curve to the pure solution curve was 0.93–1.05. The results showed the established method could provide sufficient accuracy and stability for the qualitative and quantitative determination of MF samples.

In order to highlight the sensitivity of the method, the LODs of each target compound were compared with other techniques: MLC-HSLC [27], HILIC-UHPLC-QTRAP/MS [28], and UPLC–MS/MS [16], and the results of comparison are listed in Table S1. The LOD range of our method is 0.14–5.00 ng/mL, and the LOD range of other methods is 250–410 ng/mL, 0.45–53.5 ng/mL, and 0.17–61.2 ng/mL, respectively. The LOD range of nucleoside components was 0.16–3.37 ng/mL, although few nucleoside components in other methods were available for effective comparison, but overall, this method was more sensitive than other methods, especially in arginine, alanine, and asparagine. The previous studies used the amino acid analyzer and PITC-HPLC methods [20, 21] in MF. However, both methods showed some disadvantages in resolution and analysis time, required over 90 minutes. Our solution time was 11 min included balanced column conditions, which means this analytical method should take shorter time than other methods in MF.

### 3.4. Quantitative Analyses

Contents of the 30 components in samples were determined using UFLC-MS/MS. Quantitative determination is shown in Table S2. The data from all the samples elucidated that the total contents of amino acids and nucleosides were significantly different between varieties of MF in the order Nangao (123800.00 *μ*g/g) > Daqingmei (8330.99 *μ*g/g) > Zhaoshuimei (8081.55 *μ*g/g) > Yanmei (6870.08 *μ*g/g) > Shishengmei (4622.85 *μ*g/g) > Baimei (2599.75 *μ*g/g).

In terms of individual components, aspartic acid is the most abundant amino acid in MF, which is consistent with the previous results [2]. In all the samples, the average of the aspartic acid content was 2599.41 *μ*g/g followed by *L*-asparagine, *γ*-aminobutyric acid, *L*-valine, and *L*-histidine; thymidine was the lowest (0.54 *μ*g/g). Adenosine had the highest content among the nucleoside components followed by uridine uracil, and adenine. Interestingly, the content of aspartic acid in the samples from Nangao was significantly higher than the other varieties. Aspartic acid can regulate the metabolism of the brain and nerves and is often used to treat heart disease, liver dysfunction, and hypertension [13, 14]. Adenosine, as an antiarrhythmic drug, can treat angina pectoris, myocardial infarction, cerebrovascular disorders, etc. Therefore, we speculated that the clinical, medicinal, and edible value of Nangao was higher than the other varieties.

### 3.5. Multivariate Statistical Analyses of Samples

**Figure 2:**
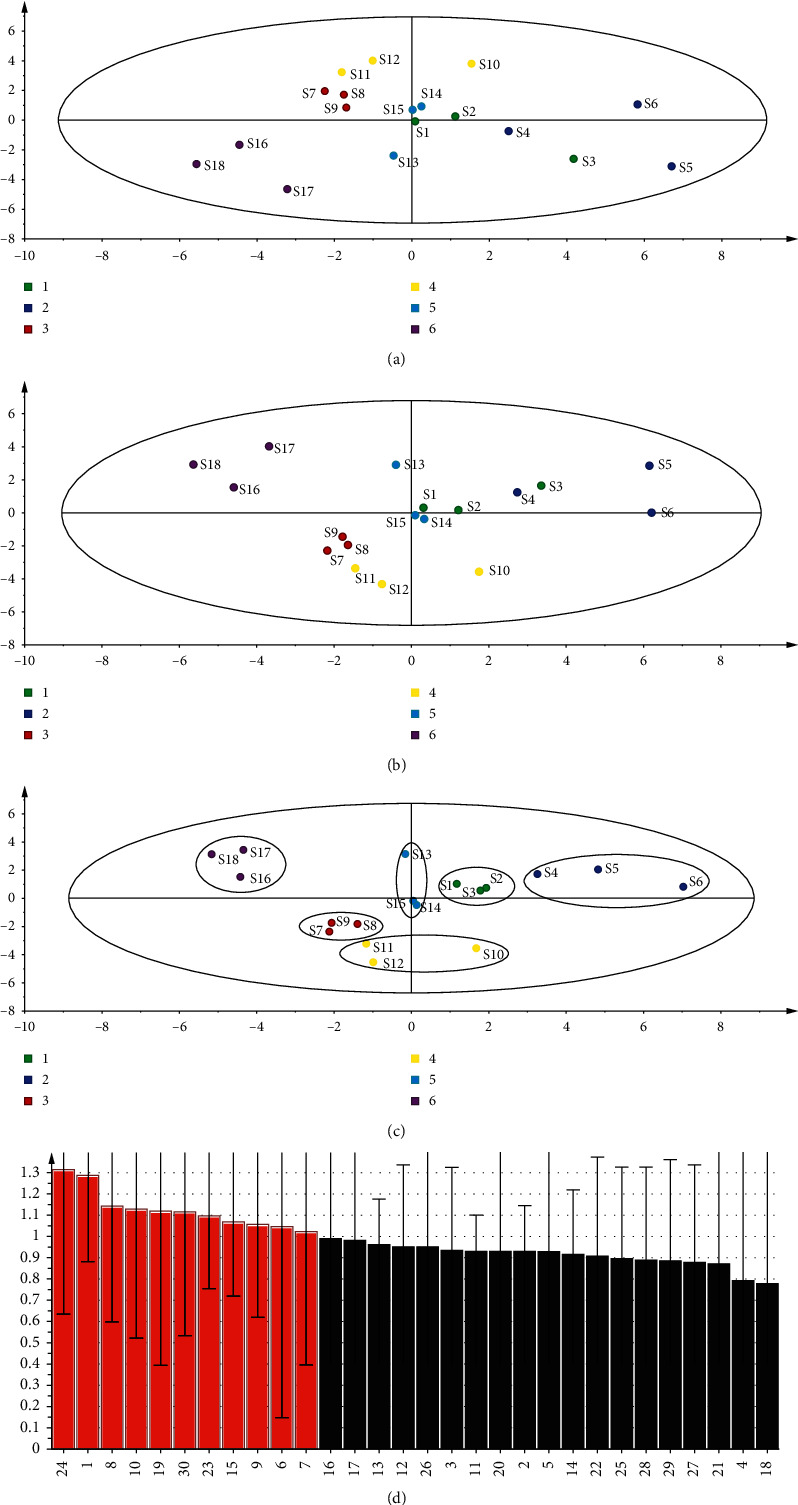
Multivariate statistical analyses of samples. (a) Principal component analysis (PCA) score chart. (b) Partial least square discriminant analysis (PLS-DA) score chart. (c) Orthogonal partial least square discriminant analysis (OPLS-DA) score chart. (d) VIP.

Analyzed data for 30 compositions of each sample are shown in Figure 2. After the original data were standardized, PCA was performed with SPSS23.0 to obtain the eigenvalues and variance contributions of principle components (PCs) [29, 30]. The results are given in Table S3. The eigenvalues of the first seven PCs were greater than 1, the cumulative contributions reached 89.04%, and the first three PCs (35.89% for PC1, 20.88% for PC2, and 11.68% for PC3) simplified the multidimensional dataset to a 3D dataset; the remaining PCs were discarded for the less effect. PC1 had good correlation with *L*-aspartic acid, *L*-asparagine, *L*-histidine, and *L*-proline. PC2 showed good correlation with *L*-valine, adenine, and hypoxanthine, and PC3 showed good correlation with glycine (Table S4). The results indicated that the aforementioned components might contribute to the classification of samples. According to the scatter plots (Figure 2(a)), PCA results showed the differences between samples but could not provide a clear classification for the samples.

PLS-DA and OPLS-DA were used to extend to discover the factors leading to different varieties [31, 32]; two methods were selected for this study. The PLS-DA results showed R2Y was 0.883, indicating that the model fitted well, but the model according to cross validation *Q*2 = 0.44 < 0.5 indicated that the model prediction result was not ideal; the score plot is shown in Figure 2(b). The OPLS-DA results showed R2Y was 0.878, indicating that the model fitted well, and *Q*2 = 0.525 > 0.5 indicated that the model predicted well. In the OPLS-DA score plot (Figure 2(c)), the samples were clearly divided into six groups; each group corresponded to the varieties of samples. In the VIP loading diagram of OPLS-DA (Figure 2(d)), the large load (VIP > 1) could be regarded as a marker component that contributed greatly to the classification of samples; *L*-isoleucine, cytidine, *L*-cystine, *L*-methionine, glycine, hypoxanthine, *L*-tyrosine, adenine, *L*-arginine, *L*-alanine, and *L*-threonine could distinguish different varieties of MF samples. These results indicated that amino acids and nucleosides might be regarded as chemical markers for MF classification.

Samples were analyzed comprehensively by TOPSIS, and ranking results were obtained. The data matrix of 30 components in six varieties was normalized, and the corresponding standardized matrix was established. *Z*_*ij*_ value was calculated according to formula (1), and the results are shown in Table S5. The optimal vector (*Z*^+^) and the worst vector (*Z*^−^) were obtained according to the normalized samples: *Z*^+^ = (0.4509, 0.4931, 0.4299, 0.4362, 0.6519, 0.5515, 0.4801, 0.6745, 0.4068, 0.4464, 0.5485, 0.6494, 0.4531, 0.5864, 0.4075, 0.4956, 0.4481, 0.4908, 0.4650, 0.5296, 0.5622, 0.4623, 0.4171, 0.4140, 0.5792, 0.9878, 0.6561, 0.5689, 0.4469, 0.4113); *Z*^−^ = (0.0222, 0.0031, 0.0225, 0.0862, 0.0304, 0.0357, 0.0475, 0.0000, 0.0502, 0.0451, 0.0000, 0.0110, 0.0350, 0.0000, 0.0169, 0.0000, 0.0463, 0.0500, 0.0000, 0.0000, 0.0509, 0.0857, 0.0864, 0.0352, 0.0074, 0.0000, 0.0244, 0.0348, 0.0760, 0.1062).

*D*_*i*_^+^, *D*_*i*_^−^, and *C*_*i*_ were calculated according to formulas (2) and (3), respectively, and the results are shown in Table 4. The evaluation objects were ranked according to *C*_*i*_. The larger the value of *C*_*i*_, the better the quality. We noted significant differences in the content between different varieties, the results above demonstrated that Nangao and Daqingmei were better qualities than other varieties of MF.

## 4. Conclusion

A rapid and sensitive UFLC-MS/MS method was established and employed to determine the components of 21 amino acids and 9 nucleosides in different varieties of MF. According to the analytical results, MF had rich amino acids and nucleosides, and aspartic acid was the most abundant amino acid in MF followed by *L*-asparagine, *γ*-aminobutyric acid, *L*-valine, and *L*-histidine. Adenosine had the highest content among nucleoside components followed by uridine, uracil, and adenine. Six varieties were well classified by PCA and OPLS-DA; 11 amino acids and nucleoside components could be used as chemical markers to classify different varieties of MF. As medicinal and edible varieties, the content and quality of Nangao and Daqingmei were higher than other varieties; the obvious difference should be caused by the artificial breeding of varieties. The present study could provide theoretical basis for further evaluation of the internal quality and control of medicinal and edible MF.

## Figures and Tables

**Table 1:** The information of the sample from different regions.

Sample number	Cultivated varieties	Cultivation region
S1	Daqingmei	Shangyu, Shaoxing, Zhejiang
S2	Daqingmei	Shangyu, Shaoxing, Zhejiang
S3	Daqingmei	Keqiao, Shaoxing, Zhejiang
S4	Nangao	Lishui, Nanjing, Jiangsu
S5	Nangao	Xishan, Suzhou, Jiangsu
S6	Nangao	Yixing, Wuxi, Jiangsu
S7	Shishengmei	Dayi, Chengdu, Sichuan
S8	Shishengmei	Pingwu, Mianyang, Sichuan
S9	Shishengmei	Baoxing, Yaan, Sichuan
S10	Yanmei	Eryuan, Dali, Yunnan
S11	Yanmei	Eryuan, Dali, Yunnan
S12	Yanmei	Eryuan, Dali, Yunnan
S13	Zhaoshuimei	Guhe, Lijiang, Yunnan
S14	Zhaoshuimei	Guhe, Lijiang, Yunnan
S15	Zhaoshuimei	Guhe, Lijiang, Yunnan
S16	Baimei	Zhaoan, Zhangzhou, Fujian
S17	Baimei	Zhaoan, Zhangzhou, Fujian
S18	Baimei	Yongtai, Fuzhou, Fujian

**Table 2:** Optimised mass spectrometry parameters for determination of 30 components.

Name	CAS no.	Formula	*t*_*R*_ (min)	[M + H]^+^(m/z)	MRM (precursor ⟶ product)	DP (V)	CE (eV)
*L*-aspartic acid	56-84-8	C_4_H_7_NO_4_	1.70	134.05	134.05/87.96	59	10
*L*-asparagine	70-47-3	C_4_H_8_N_2_O_3_	6.22	132.80	132.80/115.70	46	13
*L*-serine	56-45-1	C_3_H_7_NO_3_	6.12	106.05	106.05/59.99^a^	67	8
Glycine	56-40-6	C_2_H_5_NO_2_	5.34	76.04	76.04/30.00	73	6
*L*-alanine	56-41-7	C_3_H_7_NO_2_	4.39	90.06	90.06/44.02	79	10
*L*-glutamic acid	56-86-0	C_5_H_9_NO_4_	6.53	147.08	147.08/83.92	83	14
*L*-valine	72-18-4	C_5_H_11_NO_2_	3.38	118.09	118.09/72.06	54	10
*L*-methionine	63-68-3	C_5_H_11_NO_2_S	3.41	150.06	150.06/104.03	91	10
*L*-isoleucine	73-32-5	C_6_H_13_NO_2_	5.81	132.00	132.00/86.00	66	15
*L*-histidine	71-00-1	C_6_H_9_N_3_O_2_	6.52	156.08	156.08/110.03	95	16
*L*-arginine	74-79-3	C_6_H_14_N_4_O_2_	6.46	175.12	175.12/70.02	88	18
*L*-threonine	72-19-5	C_4_H_9_NO_3_	5.70	120.30	120.30/76.80	54	11
*L*-leucine	61-90-5	C_6_H_13_NO_2_	2.81	132.10	132.10/86.05	98	10
*L*-phenylalanine	63-91-2	C_9_H_11_NO_2_	2.84	166.10	166.10/120.05	56	14
*L*-cystine	56-89-3	C_6_H_12_N_2_O_4_S_2_	7.02	240.80	240.80/151.90	71	18
*L*-tyrosine	60-18-4	C_9_H_11_NO_3_	3.56	182.16	182.16/136.08	46	17
*L*-hydroxyproline	51-35-4	C_5_H_9_NO_3_	2.89	133.80	133.80/71.80	52	25
*L*-proline	147-85-3	C_5_H_9_NO_2_	4.13	116.07	116.07/70.02	68	10
*L*-tryptophan	73-22-3	C_11_H_12_N_2_O_2_	2.73	205.00	205.00/188.00	202	15
*γ*-Aminobutyric acid	56-12-2	C_4_H_9_NO_2_	3.14	103.70	103.70/86.90	32	14
*L*-lysine hydrochloride	10098-89-2	C_6_H_15_CIN_2_O_2_	6.52	147.00	147.00/84.00	52	24
Uracil	66-22-8	C_4_H_4_N_2_O_2_	2.44	113.04	113.04/70.00	111	21
Adenine	73-24-5	C_5_H_5_N_5_	1.93	136.06	136.06/136.06	51	24
Cytidine	65-46-3	C_9_H_13_N_3_O_5_	4.77	244.09	244.09/112.00	61	10
Inosine	58-63-9	C_10_H_12_N_4_O_5_	3.66	269.00	269.00/137.05	46	15
Vernine	118-00-3	C_10_H_13_N_5_O_5_	4.56	284.30	284.30/152.00	62	15
Thymidine	50-89-5	C_10_H_14_N2O_5_	2.39	243.10	243.10/127.07	61	13
Adenosine	58-61-7	C_10_H_13_N_5_O_4_	3.47	268.10	268.10/136.07	86	23
Uridine	58-96-8	C_9_H_12_N_2_O_6_	4.72	244.90	244.90/113.00	103	13
Hypoxanthine	68-94-0	C_5_H_4_N_4_O	1.15	137.05	137.05/137.05	51	24

**Table 3:** Regression equations, detection limits (LOD), quantitation limits (LOQ), intraday and diurnal precision, stability, repeatability, recovery, and matrix effects of 30 components.

No.	Regression equation	Linear range (*μ*g/mL)	*R* ^2^	LoD (ng/mL)	LoQ (ng/mL)	Precision (RSD%)		Stability	Repeatability	Recovery		Matrix effect
						Intraday	Interday	(RSD%, *n* = 6)	(RSD%, *n* = 6)	Mean	RSD%	
						(*n* = 6)	(*n* = 3)					
1	*y* = 9421.6*x* + 136392	0.172–17.23	0.9997	0.18	0.59	2.32	2.31	2.83	2.58	104.38	3.34	1.01
2	*y* = 810.83*x* + 56376	0.625–62.51	0.9995	0.78	2.6	2.32	2.26	2.81	2.45	95.54	2.37	1.03
3	*y* = 26896*x* + 995235	0.157–15.72	0.9996	5.00	16.67	2.17	2.79	2.22	2.21	97.39	3.69	1.05
4	*y* = 24.819*x* + 221136	0.301–22.89	0.9994	2.73	9.00	1.62	2.89	2.39	2.73	104.39	2.18	1.02
5	*y* = 3025.2*x* + 667487	0.0623–12.47	0.9993	0.14	0.18	1.96	2.38	2.87	2.57	101.54	3.55	0.96
6	*y* = 116421*x* − 47671	0.101–10.13	0.9998	0.16	0.54	1.99	2.77	1.75	2.68	96.56	2.65	0.99
7	*y* = 472881*x* − 302217	0.156–25.98	0.9993	4.69	15.63	2.31	1.99	2.61	2.21	99.61	2.01	1.03
8	*y* = 61.23*x* + 5234.1	0.00153–15.38	0.9997	0.16	0.55	3.29	2.68	2.51	2.89	97.75	2.44	0.97
9	*y* = 3025.2*x* + 667384	0.203–40.69	0.9998	2.73	9.09	2.33	2.38	2.92	2.49	98.33	3.03	0.97
10	*y* = 15.908*x* − 156.29	0.306–36.75	0.9998	1.28	4.27	2.79	1.78	1.44	2.11	98.58	2.29	0.97
11	*y* = 18.312*x* + 146822	0.301–33.16	0.9999	0.51	1.69	2.35	2.41	2.27	2.23	102.31	2.59	1.01
12	*y* = 484405*x* − 927738	4.211–420.13	0.9992	2.75	9.17	1.28	2.31	1.86	2.68	95.02	2.18	0.95
13	*y* = 431347*x* − 145124	1.847–184.73	0.9994	0.37	1.23	1.56	2.01	2.46	1.74	97.35	2.56	0.98
14	*y* = 242.73*x* + 45133	0.919–36.75	0.9996	1.85	6.17	2.05	2.89	2.34	2.74	100.57	2.67	1.02
15	*y* = 438305*x* − 224115	0.919–14.19	0.9998	3.00	10.00	2.38	2.12	2.79	2.95	96.52	2.82	0.99
16	*y* = 2745.6*x* − 132315	1.617–40.43	0.9998	0.50	1.66	2.06	2.68	1.82	2.75	97.41	2.91	0.94
17	*y* = 669.72*x* + 38649	2.281–45.62	0.9997	2.00	0.67	1.51	2.59	2.12	2.88	102.23	2.61	0.96
18	*y* = 107.22*x* + 129047	0.0169–16.94	0.9996	0.22	0.73	2.21	2.36	1.76	2.89	98.03	3.89	0.98
19	*y* = 102.67*x* + 2102.4	0.0121–10.21	0.9996	0.83	2.78	2.73	2.56	2.09	2.88	98.35	2.61	0.96
20	*y* = 5894.2*x* + 683598	0.784–15.68	0.9998	3.26	10.9	2.28	2.36	2.59	2.66	96.73	2.67	1.01
21	*y* = 1289.2*x* + 364183	0.503–150.97	0.9992	0.4	1.33	2.06	1.99	2.16	2.01	104.94	2.09	0.98
22	*y* = 294.16*x* + 20062	0.0559–50.27	0.9997	1.47	4.90	2.34	2.56	2.17	2.01	102.21	2.88	1.01
23	*y* = 34948*x* − 92208	0.168–11.68	0.9997	0.88	2.94	1.99	2.37	2.89	2.25	97.15	2.31	1.05
24	*y* = 5235.4*x* + 8471.5	0.0287–20.87	0.9996	0.16	0.54	2.14	2.78	2.03	2.01	98.45	2.51	0.97
25	*y* = 9189.8*x* − 69366	0.0534–10.68	0.9995	0.63	2.08	1.75	2.88	2.33	2.07	96.64	2.44	0.98
26	*y* = 282.98*x* + 42660	0.0102–20.43	0.9997	3.37	11.2	1.38	2.13	2.26	2.28	94.89	2.11	0.93
27	*y* = 10624*x* + 38109	0.0127–12.76	0.9998	0.80	2.67	2.38	2.34	2.86	2.46	103.21	2.61	1.03
28	*y* = 13059*x* − 240433	0.205–20.51	0.9991	0.66	2.18	1.91	3.23	1.52	2.07	99.18	2.47	0.94
29	*y* = 32.836*x* + 2686.9	0.145–30.97	0.9995	2.11	7.04	1.85	2.13	2.76	1.94	97.26	2.06	1.03
30	*y* = 22649*x* + 62656	0.0106–13.79	0.9991	0.22	1.23	2.06	1.97	2.23	2.06	103.24	2.36	1.02

**Table 4:** Sequencing of samples according to quality by TOPSIS.

Sample	*D*_*i*_^+^	*D*_*i*_^−^	*C*_*i*_	Ranking
S1	2.1051	0.9899	0.3198	10
S2	1.7748	1.4024	0.4414	4
S3	1.8297	1.5782	0.4631	3
S4	1.8716	1.1848	0.3876	5
S5	1.7277	1.7495	0.5031	1
S6	1.7289	1.6868	0.4938	2
S7	2.2416	0.9874	0.3058	11
S8	2.3036	0.6737	0.2263	17
S9	2.1677	0.8285	0.2765	13
S10	1.9502	1.1903	0.3790	7
S11	2.3375	0.6425	0.2156	18
S12	2.1381	1.1526	0.3503	9
S13	1.9987	1.2252	0.3800	6
S14	2.0088	1.0863	0.3510	8
S15	2.1371	0.9396	0.3054	12
S16	2.4264	0.7281	0.2308	16
S17	2.3657	0.8963	0.2748	14
S18	2.4811	0.8541	0.2561	15

## Data Availability

The data used to support the findings of this study are available from the corresponding author upon request.

## Supplementary Materials

Supplementary Materials Figure S1: the chemical structures of 30 amino acid and nucleotide standard compounds. Table S1: the LODs detected by UFLC-MS/MS, MLC -HSLC, HILIC-UHPLC-QTRAP/MS, and UPLC -MS/MS to analyze the sensitivity of four methods. Table S2: contents of 30 amino acids and nucleosides in samples by UFLC -MS/MS method. Table S3: eigenvalue and variance contributions of principle components (PCs). After standardizing the original data of 30 components, PCA was performed with SPSS23.0 to obtain the eigenvalues and variance contributions of PCs. Table S4: initial factor loading matrix of the first three PCs. Table S5:matrix after normalization of 30 components in samples. Using formula (1) to calculate Zij value..
