# Supplementary material for: Comparative Analysis of Free Amino Acids and Nucleosides in Different Varieties of Mume Fructus Based on Simultaneous Determination and Multivariate Statistical Analyses
Source: Int J Anal Chem. 2020 Aug 1;2020:4767605. doi: 10.1155/2020/4767605 (PMC7416269; doi:10.1155/2020/4767605)
Supplement: Supplementary Materials — Figure S1: the chemical structures of 30 amino acid and nucleotide standard compounds. Table S1: the LODs detected by UFLC-MS/MS, MLC -HSLC, HILIC-UHPLC-QTRAP/MS, and UPLC -MS/MS to analyze the sensitivity of four methods. Table S2: contents of 30 amino acids and nucleosides in samples by UFLC -MS/MS method. Table S3: eigenvalue and variance contributions of principle components (PCs). After standardizing the original data of 30 components, PCA was performed with SPSS23.0 to obtain the eigenvalues and variance contributions of PCs. Table S4: initial factor loading matrix of the first three PCs. Table S5:matrix after normalization of 30 components in samples. Using formula (1) to calculate Zij value.. [file 4767605.f1.docx]

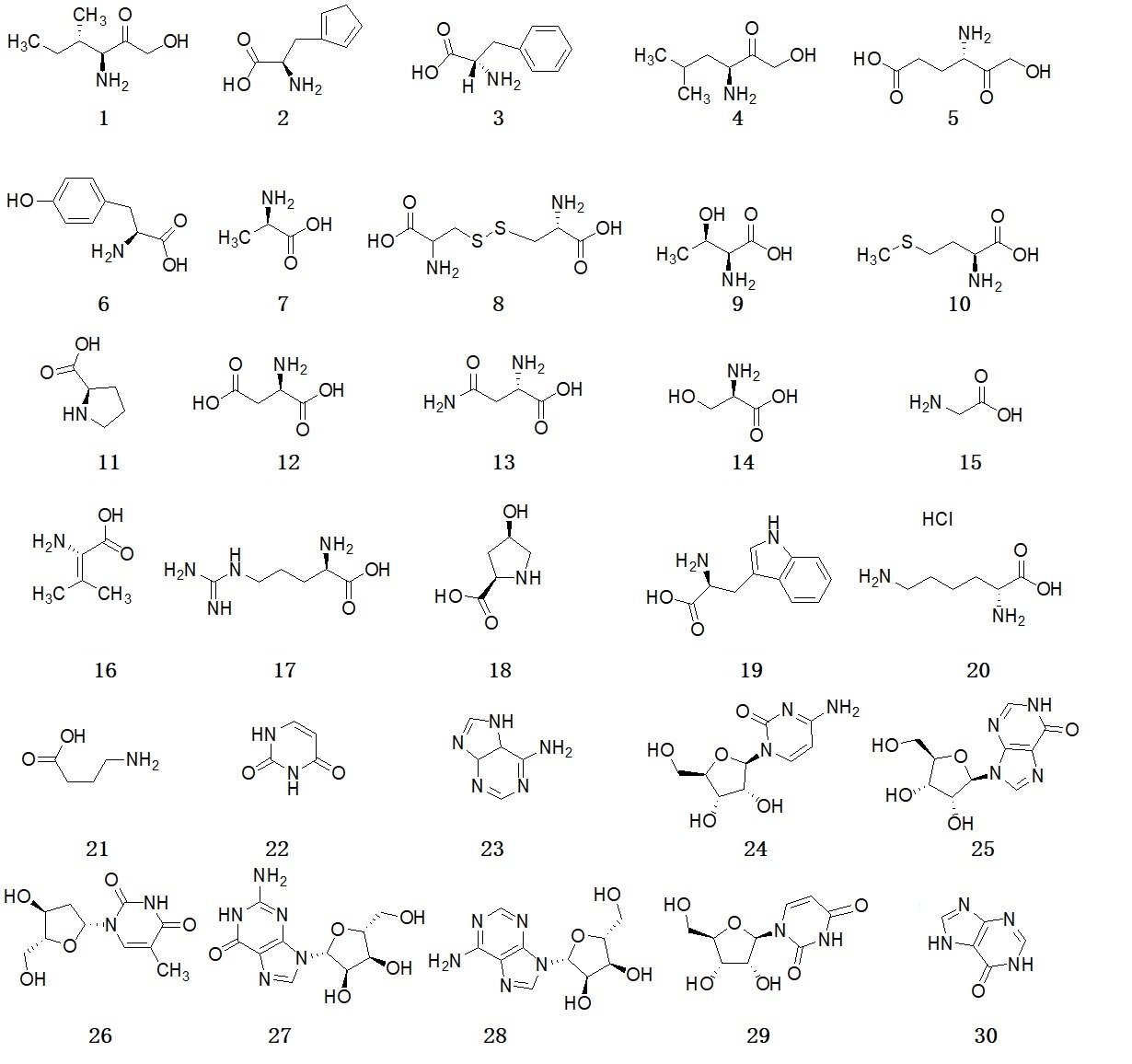


**Figure S1** Chemical structures of the 30 standard compounds

**Table S1** Comparation of LODs with other commonly used methods.

| number | LODs of analytes (ng/mL) | | | |
| --- | --- | --- | --- | --- |
|  | UFLC-MS/MS^a^ | MLC–HSLC ^b^ | HILIC-UHPLC-MS/MS^b^ | UPLC- MS/MS ^b^ |
| *L*-aspartic acid | 0.18 | 290 | 21.52 | / |
| *L* -asparagine | 0.78 | / | 28.18 | 61.20 |
| *L*-serine | 5.00 | 310 | 10.54 | / |
| glycine | 2.73 | 260 | 16.86 | / |
| *L* -alanine | 0.14 | 410 | 15.52 | 23.60 |
| *L* -glutamic acid | 0.16 | / | 35.50 | 7.30 |
| *L* -valine | 4.69 | 250 | 2.55 | 7.45 |
| *L* -methionine | 0.16 | 340 | 1.04 | 1.06 |
| *L* -isoleucine | 2.73 | 370 | 1.46 | / |
| *L* -histidine | 1.28 | 400 | 10.68 | / |
| *L* -arginine | 0.51 | 330 | 15.36 | / |
| *L* -threonine | 2.75 | 390 | 29.76 | / |
| *L* -leucine | 0.37 | 270 | 0.84 | 1.91 |
| *L* -phenylalanine | 1.85 | 340 | 0.32 | 0.61 |
| *L* -cystine | 3.00 | 290 | 20.50 | / |
| *L* -tyrosine | 0.50 | 390 | 0.35 | 5.90 |
| *L* -hydroxyproline | 2.00 | / | 1.34 | 4.53 |
| *L* -proline | 0.22 | / | 0.21 | 0.31 |
| *L* -tryptophan | 0.83 | / | 0.75 | 1.02 |
| γ-aminobutyric acid | 3.26 | / | 0.15 | 0.17 |
| *L*-lysine hydrochloride | 0.40 | 320 | / | / |
| uracil | 1.47 | / | / | / |
| adenine | 0.88 | / | / | / |
| cytidine | 0.16 | / | / | 0.57 |
| inosine | 0.63 | / | / | / |
| vernine | 3.37 | / | / | 0.68 |
| thymidine | 0.80 | / | / | 3.12 |
| adenosine | 0.66 | / | / | / |
| uridine | 2.11 | / | / | / |
| hypoxanthine | 0.22 | / | / | / |
| time | 11min | 23min | 7.5min | 16min |

^A^results obtained in this paper; ^b^results obtained from the literature including MLC–HSLC [27], HILIC-UHPLC-QTRAP/MS[28] and UPLC–MS/MS[16].

**Table S2** Contents of 30 amino acids and nucleosides in samples

| Sample | *L*-aspartic acid  (ug/g) | *L*-asparagine  (ug/g) | *L*-serine  (ug/g) | glycine  (ug/g) | *L*-alanine  (ug/g) | *L*-glutamic acid(ug/g) | *L*-valine  (ug/g) | *L*-methionine  (ug/g) | *L*-isoleucine  (ug/g) | *L*-histidine  (ug/g) |
| --- | --- | --- | --- | --- | --- | --- | --- | --- | --- | --- |
| 1 | 3942.89±44.84 | 1283.95±34.72 | 205.68±17.62 | 36.47±2.61 | 78.46±4.94 | 52.34±2.91 | 537.53±38.93 | 53.49±4.76 | 206.20±4.96 | 228.34±8.74 |
| 2 | 2534.86±68.30 | 1056.05±13.09 | 441.91±15.81 | 39.34±3.01 | 98.19±5.99 | 65.35±4.57 | 464.64±23.18 | 50.68±6.75 | 204.97±4.77 | 289.50±6.43 |
| 3 | 3192.86±49.62 | 1824.59±17.22 | 136.67±15.48 | 21.54±0.92 | 53.99±6.44 | 35.87±3.27 | 400.43±18.83 | 55.53±8.18 | 172.56±3.44 | 402.77±5.64 |
| 4 | 5841.17±31.98 | 1719.92±9.03 | 522.91±5.79 | 29.60±2.01 | 88.66±4.17 | 55.08±2.48 | 343.76±24.81 | 56.41±9.29 | 212.38±5.73 | 454.04±7.02 |
| 5 | 5421.83±46.72 | 2374.81±30.79 | 244.31±18.19 | 35.58±3.69 | 50.54±3.36 | 73.49±5.14 | 411.65±22.12 | 135.07±14.42 | 166.97±9.21 | 642.15±5.93 |
| 6 | 5923.49±64.11 | 2714.12±34.28 | 569.44±17.39 | 53.56±3.02 | 105.93±6.54 | 120.56±7.61 | 763.27±26.43 | 45.95±3.51 | 301.46±5.48 | 536.37±6.98 |
| 7 | 1456.24±53.56 | 911.53±36.81 | 307.89±19.42 | 20.49±3.55 | 172.65±2.89 | 33.27±5.86 | 720.66±22.01 | 40.31±7.12 | 138.27±3.61 | 161.92±5.10 |
| 8 | 1535.20±62.17 | 478.33±38.65 | 206.35±9.39 | 21.57±3.64 | 78.03±4.46 | 41.70±1.56 | 485.42±28.17 | 57.57±5.41 | 125.68±5.79 | 241.84±7.05 |
| 9 | 710.84±64.24 | 674.68±21.12 | 199.83±24.62 | 28.62±2.12 | 272.16±3.83 | 52.67±1.97 | 337.21±21.82 | 44.27±3.95 | 48.88±4.83 | 121.35±3.10 |
| 10 | 2781.01±65.99 | 1557.17±42.88 | 348.60±28.53 | 29.77±3.10 | 217.44±9.04 | 62.94±4.58 | 1166.99±20.02 | 47.96±4.33 | 255.62±6.01 | 447.7±8.17 |
| 11 | 1523.89±29.09 | 428.66±18.48 | 367.06±17.67 | 17.44±1.73 | 406.83±4.00 | 38.90±1.12 | 988.82±14.48 | 40.06±2.09 | 96.37±7.16 | 117.53±3.86 |
| 12 | 1456.52±39.11 | 280.53±36.89 | 310.87±28.63 | 23.52±1.88 | 497.43±6.41 | 29.57±1.06 | 778.36±44.55 | 54.83±2.51 | 126.47±8.32 | 308.92±4.68 |
| 13 | 2770.43±23.51 | 1102.55±23.61 | 309.44±24.95 | 86.48±5.24 | 115.58±7.18 | 35.25±4.28 | 550.25±26.24 | 623.23±11.15 | 380.36±6.03 | 271.50±4.41 |
| 14 | 2965.31±45.28 | 1293.36±26.78 | 499.19±19.58 | 68.95±2.60 | 203.97±6.01 | 30.47±1.88 | 790.47±23.05 | 355.27±8.88 | 344.69±7.17 | 486.59±4.99 |
| 15 | 2750.11±19.16 | 1031.95±35.89 | 301.46±17.72 | 57.20±3.31 | 197.75±3.45 | 53.91±4.91 | 568.17±25.63 | 238.42±1.88 | 433.95±5.33 | 348.1±6.28 |
| 16 | 353.95±22.84 | 174.72±5.58 | 46.97±10.64 | 50.63±5.98 | 33.76±5.37 | 8.76±1.79 | 116.14±18.98 | 260.49±6.44 | 108.74±5.81 | 111.93±4.45 |
| 17 | 1512.97±49.18 | 343.28±4.43 | 31.76±4.40 | 50.50±4.92 | 23.37±0.16 | 7.86±1.29 | 152.11±27.90 | 416.08±11.63 | 79.43±1.96 | 76.84±4.44 |
| 18 | 294.47±45.81 | 233.05±9.08 | 54.76±7.63 | 84.09±6.95 | 33.02±0.25 | 8.70±1.07 | 167.43±17.45 | 224.64±10.88 | 60.04±2.33 | 63.94±6.15 |

| Sample | *L*-lysine hydrochloride  (ug/g) | *L*-arginine  (ug/g) | *L*-threonine  (ug/g) | *L*-leucine  (ug/g) | *L*-phenylalanine  (ug/g) | *L*-cystine  (ug/g) | *L*-tyrosine  (ug/g) | *L*-hydroxyproline  (ug/g) | *L*-proline  (ug/g) | *L*-tryptophan  (ug/g) |
| --- | --- | --- | --- | --- | --- | --- | --- | --- | --- | --- |
| 1 | 43.65±5.05 | 173.46±7.12 | 27.33±0.68 | 19.53±1.87 | 95.44±3.05 | 20.44±1.19 | 4.5±0.60 | 1.67±0.46 | 307.78±6.54 | 2.29±0.08 |
| 2 | 55.15±5.90 | 182.91±3.55 | 30.12±1.43 | 22.06±2.34 | 109.50±5.39 | 15.46±1.05 | 5.38±1.39 | 0.17±0.05 | 401.54±6.86 | 2.46±0.19 |
| 3 | 36.04±4.74 | 107.32±5.01 | 22.95±4.39 | 16.5±1.15 | 152.02±10.16 | 25.17±1.56 | 11.82±2.11 | 1.4±0.25 | 441.31±5.83 | 5.56±0.10 |
| 4 | 47.92±5.40 | 268.77±4.70 | 17.28±1.85 | 10.51±0.72 | 155.39±8.35 | 8.01±0.56 | 3.78±0.75 | 0.84±0.05 | 444.99±6.71 | 12.53±0.16 |
| 5 | 62.98±4.41 | 280.84±7.84 | 19.89±2.32 | 2.64±0.22 | 245.58±6.32 | 23.51±1.74 | 5.73±1.54 | 0.53±0.066 | 633.79±6.65 | 14.52±0.33 |
| 6 | 103.16±4.51 | 620.63± 6.14 | 29.56±1.29 | - | 152.45±11.66 | 20.88±1.07 | 8.31±1.24 | 1.04±0.070 | 586.46±8.01 | 9.78±0.10 |
| 7 | 29.41±1.85 | 39.79±3.65 | 17.04±2.05 | 44.74±3.26 | 114.18±5.58 | 9.26±0.62 | 29.75±1.72 | 0.56±0.062 | 73.99±6.97 | 8.14±0.15 |
| 8 | 38.76±2.60 | 75.13±4.87 | 40.60±2.26 | 17.10±4.45 | 143.15±5.52 | 9.63±0.85 | 7.47±1.18 | 0.66±0.10 | 107.44±4.99 | 0.33±0.052 |
| 9 | 44.92±3.71 | 110.98±7.66 | 27.85±1.47 | 20.75±1.51 | 30.32±2.74 | 10.45±0.94 | 12.95±1.89 | 0.20±0.015 | 242.52±6.44 | 3.99±0.11 |
| 10 | 55.03±6.16 | 154.25±5.05 | 39.15±1.15 | 23.24±1.39 | 214.62±5.85 | - | 22.31±2.27 | 0.47±0.021 | 379.78±8.33 | 7.33±0.33 |
| 11 | 33.06±2.85 | 348.22±5.53 | 26.94±1.48 | 22.89±1.27 | 116.01±7.93 | - | 10.67±1.26 | 0.42±0.012 | 98.04±5.38 | 1.01±0.051 |
| 12 | 26.98±4.19 | 369.37±6.66 | 43.63±1.82 | 28.61±2.12 | 223.72±4.46 | 0.31±0.007 | 11.62±2.20 | 0.40±0.031 | 86.58±5.55 | 0.13±0.041 |
| 13 | 30.20±2.74 | 179.21±6.81 | 4.50±0.55 | 17.75±1.33 | 117.58±10.26 | 16.85±1.70 | 4.47±1.32 | 1.37±0.025 | 260.47±8.67 | 6.85±0.38 |
| 14 | 26.75±2.64 | 208.03±7.62 | 4.39±0.43 | 31.91±2.39 | 166.45±7.09 | - | 9.27±1.46 | 0.26±0.041 | 328.07±9.78 | 4.36±0.24 |
| 15 | 47.46±4.26 | 219.85±7.23 | 4.06±0.11 | 22.82±0.98 | 152.51±8.21 | - | 10.53±2.30 | 0.41±0.020 | 253.40±6.04 | - |
| 16 | 9.99±1.46 | 26.49±4.03 | 3.64±0.52 | 46.93±2.62 | 37.33±3.60 | 9.83±1.57 | 4.14±0.38 | 0.51±0.071 | 60.26±6.51 | 1.49±0.075 |
| 17 | 8.82±3.29 | 24.51±3.24 | 4.48±0.58 | 27.21±2.20 | 41.89±1.63 | - | 2.66±0.28 | 0.30±0.031 | - | 4.58±0.18 |
| 18 | 8.81±1.30 | 14.78±4.24 | 4.83±0.73 | 75.46±1.89 | 10.38±0.75 | 4.64±0.44 | 2.26±0.28 | 0.76±0.064 | 12.42±1.78 | - |

| Sample | *γ*-aminobutyric acid  (ug/g) | uracil  (ug/g) | adenine  (ug/g) | cytidine  (ug/g) | inosine  (ug/g) | thymidine  (ug/g) | guanosine  (ug/g) | adenosine  (ug/g) | uridine  (ug/g) | hypoxanthine  (ug/g) |
| --- | --- | --- | --- | --- | --- | --- | --- | --- | --- | --- |
| 1 | 786.54±4.81 | 16.76±0.30 | 24.66±0.65 | 1.87±0.095 | 2.58±0.095 | - | 0.14±0.015 | 14.38±0.73 | 19.54±0.76 | 3.11±0.11 |
| 2 | 688.55±6.47 | 32.48±0.33 | 23.61±0.84 | 1.67±0.11 | 4.78±0.47 | 8.1±0.64 | 0.59±0.032 | 25.43±0.56 | 36.54±0.93 | 2.82±0.10 |
| 3 | 2575.81±5.66 | 35.67±0.21 | 21.77±0.82 | 1.47±0.19 | 13.61±1.08 | - | 2.44±0.062 | 77.52±1.16 | 57.67±1.22 | 2.95±0.17 |
| 4 | 712.49±5.68 | 19.34±0.39 | 33.09±0.97 | 0.63±0.061 | 4.66±0.59 | - | 0.57±0.060 | 28.13±0.90 | 28.52±0.99 | 4.52±0.18 |
| 5 | 1272.12±7.86 | 46.25±1.11 | 29.63±0.94 | 0.25±0.04 | 13.19±0.71 | - | 2.39±0.17 | 76.55±1.31 | 59.09±1.39 | 4.36±0.17 |
| 6 | 1049.00±8.19 | 27.54±1.24 | 26.45±1.26 | 1.89±0.092 | 4.68±0.51 | - | 0.76±0.062 | 27.54±1.17 | 31.23±1.29 | 3.26±0.075 |
| 7 | 413.29±11.87 | 16.06±0.31 | 19.55±0.21 | 6.07±3.12 | 1.2±0.14 | - | 0.42±0.056 | 6.48±0.52 | 14.51±1.07 | 3.12±0.20 |
| 8 | 719.87±9.13 | 10.72±0.56 | 17.80±0.72 | 6.23±4.57 | 2.55±0.22 | - | 0.22±0.01 | 15.07±0.33 | 15.65±1.20 | 2.23±0.11 |
| 9 | 1472.37±7.10 | 16.10±0.83 | 16.87±0.46 | 6.94±5.09 | 3.53±0.33 | - | 0.41±0.02 | 16.91±0.53 | 24.63±1.58 | 4.58±0.20 |
| 10 | 1047.60±9.60 | 10.36±0.88 | 11.73±0.86 | 0.23±0.04 | 3.76±0.28 | 1.17±0.090 | 0.51±0.02 | 23.67±1.01 | 14.30±1.21 | 1.69±011 |
| 11 | 928.21±7.02 | 15.08±0.17 | 19.04±0.77 | 1.79±0.16 | 1.61±0.15 | - | 0.16±0.03 | 8.56±0.13 | 10.78±1.77 | 2.54±0.15 |
| 12 | 1284.43±11.70 | 17.29±0.97 | 9.48±0.84 | 2.47±0.075 | 3.39±0.37 | 0.51±0.025 | 0.10±0.032 | 19.29±0.81 | 17.73±1.89 | 1.51±0.15 |
| 13 | 581.44±8.14 | 18.12±0.57 | 34.38±1.01 | 0.98±0.11 | 2.78±0.39 | - | 0.22±0.03 | 17.53±0.75 | 28.37±1.69 | 4.60±0.28 |
| 14 | 1058.39±6.03 | 18.42±0.79 | 14.02±0.70 | 0.72±0.062 | 4.1±0.56 | - | 0.31±0.047 | 26.57±1.25 | 25.66±0.94 | 1.98±0.18 |
| 15 | 906.55±8.65 | 17.27±0.89 | 19.95±0.46 | 0.58±0.036 | 4.62±0.24 | - | 0.26±0.015 | 28.58±0.96 | 26.40±1.32 | 2.41±0.16 |
| 16 | 816.31±9.50 | 8.71±0.38 | 34.46±1.31 | 2.72±0.062 | 0.17±0.02 | - | 0.2±0.036 | 21.64±1.14 | 15.4±0.91 | 2.30±0.16 |
| 17 | 764.36±11.50 | 38.40±1.12 | 46.75±1.59 | 1.28±0.060 | 3.55±0.20 | - | 0.21±0.025 | 20.62±1.18 | 55.24±2.09 | 6.10±0.20 |
| 18 | 233.47±4.12 | 15.65±0.73 | 37.61±1.03 | 1.66±0.074 | 0.86±0.70 | - | 0.23±0.025 | 4.52±0.35 | 18.53±0.88 | 5.00±0.17 |

**Table S3 Eigenvalue of principal component and contribution rate**

| Principal component | Initial Eigenvalues | | | Eigenvalues after rotation | | |
| --- | --- | --- | --- | --- | --- | --- |
|  | Total | Variance  (%) | - Accumulative contribution rate（%） | Total | Variance  (%) | - Accumulative contribution rate（%） |
| 1 | 10.769 | 35.898 | 35.898 | 10.769 | 35.898 | 35.898 |
| 2 | 6.262 | 20.875 | 56.773 | 6.262 | 20.875 | 56.773 |
| 3 | 3.503 | 11.675 | 68.448 | 3.503 | 11.675 | 68.448 |
| 4 | 2.177 | 7.257 | 75.705 | 2.177 | 7.257 | 75.705 |
| 5 | 1.615 | 5.383 | 81.089 | 1.615 | 5.383 | 81.089 |
| 6 | 1.268 | 4.225 | 85.314 | 1.268 | 4.225 | 85.314 |
| 7 | 1.120 | 3.734 | 89.048 | 1.120 | 3.734 | 89.048 |

**Table S4 Initial factor loading matrix**

| component | PC1 | PC2 | PC3 |
| --- | --- | --- | --- |
| L-aspartic acid | 0.88 | 0.09 | 0.34 |
| L-asparagine | 0.95 | 0.07 | 0.18 |
| L-serine | 0.56 | -0.52 | 0.48 |
| glycine | -0.22 | 0.45 | 0.70 |
| L-alanine | -0.10 | -0.79 | -0.15 |
| L-glutamic acid | 0.80 | -0.27 | 0.25 |
| L-valine | 0.29 | 0.81 | 0.14 |
| L-methionine | -0.30 | 0.55 | 0.43 |
| L-isoleucine | 0.44 | -0.12 | 0.64 |
| L-histidine | 0.91 | -0.08 | 0.16 |
| L-lysine hydrochloride | 0.81 | -0.29 | 0.22 |
| L-arginine | 0.62 | -0.39 | 0.31 |
| L-threonine | 0.31 | -0.64 | -0.38 |
| L-leucine | -0.82 | 0.07 | -0.05 |
| L-Phenylalanine | 0.72 | -0.40 | -0.06 |
| L-cystine | 0.57 | 0.40 | -0.12 |
| L-tyrosine | 0.05 | -0.59 | -0.29 |
| L-hydroxyproline | 0.24 | 0.30 | 0.12 |
| L-proline | 0.95 | 0.08 | 0.13 |
| L-tryptophan | 0.70 | 0.26 | 0.16 |
| γ-aminobutyric acid | 0.52 | 0.03 | -0.64 |
| uracil | 0.57 | 0.57 | -0.23 |
| adenine | -0.20 | 0.86 | 0.27 |
| cytidine | -0.43 | -0.14 | -0.20 |
| inosine | 0.80 | 0.35 | -0.41 |
| thymidine | 0.10 | -0.10 | -0.01 |
| vernine | 0.73 | 0.40 | -0.48 |
| adenosine | 0.74 | 0.40 | -0.42 |
| uridine | 0.54 | 0.70 | -0.25 |
| hypoxanthine | -0.10 | 0.86 | 0.14 |

**Table S5 Matrix after normalization of 30 components in different varieties of Fructus mume**

| NO. | Z | | | | | | | | | |
| --- | --- | --- | --- | --- | --- | --- | --- | --- | --- | --- |
|  | *L*-aspartic acid | *L*-asparagine | *L*-serine | glycine | *L*-alanine | *L*-glutamic acid | *L*-valine | *L*-methionine | *L*-isoleucine | *L*-histidine |
| S1 | 0.3000 | 0.2329 | 0.1577 | 0.1823 | 0.1027 | 0.2349 | 0.2210 | 0.0582 | 0.2203 | 0.1591 |
| S2 | 0.1929 | 0.1917 | 0.3300 | 0.1994 | 0.1281 | 0.2972 | 0.1911 | 0.0551 | 0.2200 | 0.2007 |
| S3 | 0.2429 | 0.3317 | 0.1027 | 0.1052 | 0.0710 | 0.1619 | 0.1645 | 0.0589 | 0.1817 | 0.2801 |
| S4 | 0.4445 | 0.3117 | 0.3933 | 0.1512 | 0.1160 | 0.2427 | 0.1414 | 0.0616 | 0.2260 | 0.3154 |
| S5 | 0.3991 | 0.4312 | 0.1825 | 0.1806 | 0.0643 | 0.3357 | 0.1692 | 0.1469 | 0.1784 | 0.4464 |
| S6 | 0.4509 | 0.4931 | 0.4299 | 0.2688 | 0.1389 | 0.5515 | 0.3139 | 0.0491 | 0.3205 | 0.3733 |
| S7 | 0.1109 | 0.1660 | 0.2312 | 0.1012 | 0.2267 | 0.1471 | 0.2962 | 0.0442 | 0.1438 | 0.1121 |
| S8 | 0.1167 | 0.0881 | 0.1531 | 0.1064 | 0.0990 | 0.1873 | 0.2005 | 0.0621 | 0.1348 | 0.1682 |
| S9 | 0.0540 | 0.1230 | 0.1500 | 0.1350 | 0.3544 | 0.2394 | 0.1381 | 0.0481 | 0.0502 | 0.0841 |
| S10 | 0.2118 | 0.2831 | 0.2637 | 0.1505 | 0.2823 | 0.2868 | 0.4801 | 0.0521 | 0.2742 | 0.3114 |
| S11 | 0.1182 | 0.0774 | 0.1276 | 0.0862 | 0.2690 | 0.1730 | 0.1964 | 0.0430 | 0.1029 | 0.0819 |
| S12 | 0.1109 | 0.0510 | 0.2312 | 0.1200 | 0.6519 | 0.1337 | 0.3194 | 0.0595 | 0.1359 | 0.2149 |
| S13 | 0.2108 | 0.2009 | 0.2294 | 0.4362 | 0.1512 | 0.1623 | 0.2263 | 0.6745 | 0.4068 | 0.1881 |
| S14 | 0.2254 | 0.2345 | 0.3728 | 0.3505 | 0.2690 | 0.1376 | 0.3249 | 0.3821 | 0.3658 | 0.3389 |
| S15 | 0.2095 | 0.1854 | 0.2249 | 0.2889 | 0.2586 | 0.2461 | 0.2336 | 0.0000 | 0.4637 | 0.2425 |
| S16 | 0.0272 | 0.0031 | 0.0269 | 0.2482 | 0.0495 | 0.0409 | 0.0475 | 0.2819 | 0.1141 | 0.0779 |
| S17 | 0.1155 | 0.0621 | 0.0225 | 0.2570 | 0.0304 | 0.0357 | 0.0630 | 0.4539 | 0.0852 | 0.0535 |
| S18 | 0.0222 | 0.0043 | 0.0281 | 0.4239 | 0.0433 | 0.0404 | 0.0691 | 0.2443 | 0.0643 | 0.0451 |

| NO. | Z | | | | | | | | | |
| --- | --- | --- | --- | --- | --- | --- | --- | --- | --- | --- |
|  | *L*-lysine hydrochloride | *L*-arginine | *L*-threonine | *L*-leucine | *L*-phenylalanine | *L*-cystine | *L*-tyrosine | *L*-hydroxyproline | *L*-proline | *L*-tryptophan |
| S1 | 0.2337 | 0.1795 | 0.2624 | 0.1525 | 0.1585 | 0.3695 | 0.0896 | 0.4908 | 0.2250 | 0.0850 |
| S2 | 0.2876 | 0.1916 | 0.2846 | 0.1533 | 0.1802 | 0.2917 | 0.0928 | 0.0500 | 0.2939 | 0.0898 |
| S3 | 0.1851 | 0.1116 | 0.2152 | 0.1309 | 0.2556 | 0.4607 | 0.2381 | 0.4379 | 0.3223 | 0.2056 |
| S4 | 0.2490 | 0.2806 | 0.1636 | 0.0769 | 0.2579 | 0.1440 | 0.0756 | 0.2469 | 0.3263 | 0.4650 |
| S5 | 0.3292 | 0.2927 | 0.1843 | 0.0224 | 0.4075 | 0.4069 | 0.1188 | 0.1528 | 0.4650 | 0.5296 |
| S6 | 0.5485 | 0.6494 | 0.2938 | 0.0000 | 0.2481 | 0.4123 | 0.1751 | 0.2968 | 0.4296 | 0.3618 |
| S7 | 0.1555 | 0.0412 | 0.1556 | 0.3501 | 0.1898 | 0.0000 | 0.6074 | 0.1705 | 0.0489 | 0.3006 |
| S8 | 0.1908 | 0.0777 | 0.3834 | 0.1345 | 0.2394 | 0.1729 | 0.1461 | 0.1822 | 0.0783 | 0.0115 |
| S9 | 0.2367 | 0.1142 | 0.2580 | 0.1742 | 0.0514 | 0.2099 | 0.2899 | 0.0588 | 0.1778 | 0.1484 |
| S10 | 0.2853 | 0.1565 | 0.3739 | 0.1843 | 0.3572 | 0.0000 | 0.4481 | 0.1411 | 0.2775 | 0.2809 |
| S11 | 0.1775 | 0.0506 | 0.2547 | 0.1896 | 0.1896 | 0.0000 | 0.2182 | 0.1264 | 0.0717 | 0.0371 |
| S12 | 0.1375 | 0.3855 | 0.4531 | 0.2233 | 0.3710 | 0.0053 | 0.2381 | 0.1146 | 0.0000 | 0.0045 |
| S13 | 0.1590 | 0.1873 | 0.0389 | 0.1349 | 0.1925 | 0.3108 | 0.0861 | 0.4085 | 0.1907 | 0.2576 |
| S14 | 0.1377 | 0.2157 | 0.0386 | 0.2491 | 0.2772 | 0.0000 | 0.1871 | 0.0794 | 0.2385 | 0.1596 |
| S15 | 0.2455 | 0.2306 | 0.0381 | 0.1859 | 0.2511 | 0.0000 | 0.2072 | 0.1205 | 0.1853 | 0.0000 |
| S16 | 0.0473 | 0.0243 | 0.0350 | 0.3657 | 0.0607 | 0.1804 | 0.0821 | 0.1665 | 0.0442 | 0.0549 |
| S17 | 0.0000 | 0.0221 | 0.0388 | 0.2156 | 0.0699 | 0.0000 | 0.0498 | 0.0911 | 0.0000 | 0.1648 |
| S18 | 0.0449 | 0.0110 | 0.0441 | 0.5864 | 0.0169 | 0.0823 | 0.0463 | 0.2322 | 0.0088 | 0.0000 |

| NO. | Z | | | | | | | | | |
| --- | --- | --- | --- | --- | --- | --- | --- | --- | --- | --- |
|  | *γ*-aminobutyric acid | uracil | adenine | cytidine | inosine | thymidine | guanosine | adenosine | uridine | hypoxanthine |
| S1 | 0.1717 | 0.1693 | 0.2206 | 0.2848 | 0.1115 | 0.0000 | 0.0380 | 0.1046 | 0.1477 | 0.2084 |
| S2 | 0.1506 | 0.3259 | 0.2083 | 0.2512 | 0.2000 | 0.9878 | 0.1573 | 0.1886 | 0.2765 | 0.1916 |
| S3 | 0.5622 | 0.3572 | 0.2022 | 0.2205 | 0.5792 | 0.0000 | 0.6561 | 0.5689 | 0.4330 | 0.1963 |
| S4 | 0.1555 | 0.1921 | 0.2997 | 0.0995 | 0.2152 | 0.0000 | 0.1573 | 0.2110 | 0.2145 | 0.3072 |
| S5 | 0.2777 | 0.4623 | 0.2664 | 0.0398 | 0.5653 | 0.0000 | 0.6317 | 0.5613 | 0.4469 | 0.2904 |
| S6 | 0.2287 | 0.2761 | 0.2379 | 0.2925 | 0.2065 | 0.0000 | 0.2115 | 0.2003 | 0.2374 | 0.2198 |
| S7 | 0.0901 | 0.1604 | 0.1733 | 0.3783 | 0.0512 | 0.0000 | 0.1112 | 0.0507 | 0.1051 | 0.2144 |
| S8 | 0.1580 | 0.1081 | 0.1542 | 0.1470 | 0.1093 | 0.0000 | 0.0569 | 0.1096 | 0.1170 | 0.1479 |
| S9 | 0.3212 | 0.1621 | 0.1466 | 0.1623 | 0.1584 | 0.0000 | 0.1166 | 0.1231 | 0.1801 | 0.3065 |
| S10 | 0.2284 | 0.1100 | 0.1068 | 0.0352 | 0.1636 | 0.1427 | 0.1383 | 0.1712 | 0.1060 | 0.1116 |
| S11 | 0.2025 | 0.1517 | 0.1707 | 0.2603 | 0.0677 | 0.0000 | 0.0434 | 0.0619 | 0.0760 | 0.1714 |
| S12 | 0.2797 | 0.1732 | 0.0864 | 0.3783 | 0.1406 | 0.0622 | 0.0244 | 0.1428 | 0.1303 | 0.1062 |
| S13 | 0.1268 | 0.1806 | 0.3098 | 0.1525 | 0.1215 | 0.0000 | 0.0596 | 0.1263 | 0.2166 | 0.3065 |
| S14 | 0.2313 | 0.1918 | 0.1238 | 0.1081 | 0.1835 | 0.0000 | 0.0786 | 0.1920 | 0.1894 | 0.1290 |
| S15 | 0.1973 | 0.1802 | 0.1753 | 0.0825 | 0.2013 | 0.0000 | 0.0705 | 0.2114 | 0.1962 | 0.1579 |
| S16 | 0.1779 | 0.0857 | 0.3083 | 0.4140 | 0.0074 | 0.0000 | 0.0461 | 0.1585 | 0.1125 | 0.1553 |
| S17 | 0.1666 | 0.3851 | 0.4171 | 0.1868 | 0.1497 | 0.0000 | 0.0569 | 0.1518 | 0.4117 | 0.4113 |
| S18 | 0.0509 | 0.1560 | 0.3396 | 0.2426 | 0.0369 | 0.0000 | 0.0624 | 0.0348 | 0.1324 | 0.3327 |
